# Deciphering complex metabolite mixtures by unsupervised and supervised substructure discovery and semi-automated annotation from MS/MS spectra†

**DOI:** 10.1039/c8fd00235e

**Published:** 2019-01-29

**Authors:** Simon Rogers, Cher Wei Ong, Joe Wandy, Madeleine Ernst, Lars Ridder, Justin J. J. van der Hooft

**Affiliations:** a School of Computing Science, University of Glasgow Glasgow UK; b Glasgow Polyomics, University of Glasgow Glasgow UK; c Collaborative Mass Spectrometry Innovation Center, Skaggs School of Pharmacy and Pharmaceutical Sciences, University of California San Diego La Jolla CA USA; d Skaggs School of Pharmacy and Pharmaceutical Sciences, University of California, San Diego San Diego California USA; e Netherlands eScience Center Amsterdam The Netherlands; f Bioinformatics Group, Wageningen University Wageningen The Netherlands justin.vanderhooft@wur.nl

## Abstract

Complex metabolite mixtures are challenging to unravel. Mass spectrometry (MS) is a widely used and sensitive technique for obtaining structural information of complex mixtures. However, just knowing the molecular masses of the mixture’s constituents is almost always insufficient for confident assignment of the associated chemical structures. Structural information can be augmented through MS fragmentation experiments whereby detected metabolites are fragmented, giving rise to MS/MS spectra. However, how can we maximize the structural information we gain from fragmentation spectra? We recently proposed a substructure-based strategy to enhance metabolite annotation for complex mixtures by considering metabolites as the sum of (bio)chemically relevant moieties that we can detect through mass spectrometry fragmentation approaches. Our MS2LDA tool allows us to discover – unsupervised – groups of mass fragments and/or neutral losses, termed Mass2Motifs, that often correspond to substructures. After manual annotation, these Mass2Motifs can be used in subsequent MS2LDA analyses of new datasets, thereby providing structural annotations for many molecules that are not present in spectral databases. Here, we describe how additional strategies, taking advantage of (i) combinatorial *in silico* matching of experimental mass features to substructures of candidate molecules, and (ii) automated machine learning classification of molecules, can facilitate semi-automated annotation of substructures. We show how our approach accelerates the Mass2Motif annotation process and therefore broadens the chemical space spanned by characterized motifs. Our machine learning model used to classify fragmentation spectra learns the relationships between fragment spectra and chemical features. Classification prediction on these features can be aggregated for all molecules that contribute to a particular Mass2Motif and guide Mass2Motif annotations. To make annotated Mass2Motifs available to the community, we also present MotifDB: an open database of Mass2Motifs that can be browsed and accessed programmatically through an Application Programming Interface (API). MotifDB is integrated within ms2lda.org, allowing users to efficiently search for characterized motifs in their own experiments. We expect that with an increasing number of Mass2Motif annotations available through a growing database, we can more quickly gain insight into the constituents of complex mixtures. This will allow prioritization towards novel or unexpected chemistries and faster recognition of known biochemical building blocks.

## Abstract 2

Integration of MS2LDA substructure discovery with MAGMa spectral annotations and ClassyFire term predictions complemented with MotifDB significantly advances metabolite annotation.

## Graphical abstract

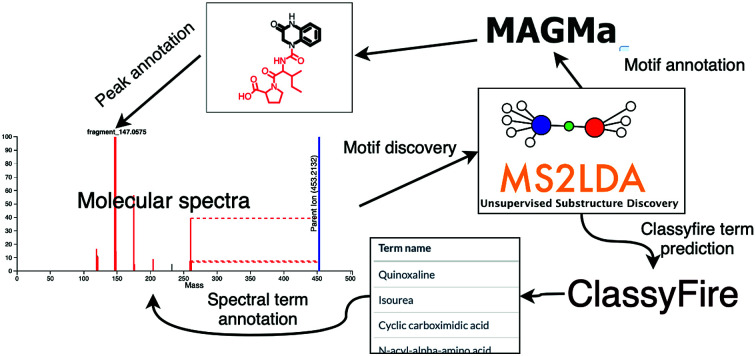

## Introduction

Complex natural mixtures are full of specialized metabolites with diverse structures and functions.^1^ In untargeted metabolomics approaches, these molecules give rise to information-rich mass spectral data sets and a key challenge is the interpretation of this data, particularly in terms of identifying chemical structures.^2,3^ This process is commonly referred to as metabolite annotation and identification,^4^ a highly challenging process that typically enables the assignment of chemical structures to only a very small percentage of the molecules detected.^2,5–7^ Consequently, the rapid and automated identification of chemical structures is one of the main obstacles hindering the discovery of novel bioactive molecules addressing global health care threats, such as antimicrobial resistance, cancer or inflammatory diseases.

Recently, we demonstrated how the unsupervised decomposition of fragment (MS2) spectra could aid in the annotation of molecules *via* identifying common fragment and loss patterns that were indicative of particular substructures (termed Mass2Motifs).^8^ We showed that through Mass2Motif discovery, we can assign substructures to more than 70% of the fragmented molecules in beer extracts and our approach (MS2LDA) is publicly available through a web application (ms2lda.org).^8^ Another widely used tool to organize fragmentation spectra is mass spectral Molecular Networking.^9,10^ In combination or as a stand-alone tool, these similarity-based fragment spectra grouping algorithms are the current state-of-the-art in untargeted metabolomics for rapidly obtaining a comprehensive overview of molecular diversity in samples.^11–15^ To retrieve chemical structural information for acquired experimental spectra, MS2 fragmentation patterns are matched directly to library reference data or *in silico* by matching substructures of candidate structures,^5,16–18^ however only a very low percentage of the molecular features (typically 2–5%, but up to 30% in rare cases) can be confidently assigned to known chemical structures. In comparison to the structural annotation of entire molecules, structural annotation of the Mass2Motifs is more straightforward and less complex, as Mass2Motifs represent smaller substructures. However, the structural annotation of Mass2Motifs is currently performed *via* a combination of manual peak searching in MS/MS databases such as MetLin^19^ and MzCloud^20^ as well as expert knowledge, and thus still represents a tedious and time-consuming step, especially for large-scale high-throughput experiments with several hundred discovered Mass2Motifs per experiment. As we and others have shown,^8,17,21,22^ the use of reference MS/MS spectra of standards speeds up the annotation process; however, with the increasing size of publicly available MS/MS reference libraries,^9,17^ complete manual Mass2Motif annotation and curation is rapidly becoming impractical. Furthermore, with the expected increase in publicly available experimental MS/MS data, the amount of structurally novel Mass2Motifs is expected to steadily rise. This will make structural predictions for Mass2Motifs of non-standards and effective reuse of previously annotated Mass2Motifs essential. Thus, the next step is to semi-automate Mass2Motif annotation and store annotated Mass2Motifs such that they can be used in the future.

In recent years, algorithms that propose chemical substructures and candidate structures for mass features have become available.^23–26^ For example, MAGMa maps possible candidate molecules to MS/MS spectra in experimental data by assigning possible substructures from a candidate molecule to the mass fragments, and subsequently ranks different candidate molecules using those annotations based on a relatively simple scoring algorithm.^27^ A complementary strategy towards structural annotation is to predict molecular properties such as fingerprints or classification based on spectral features.^28,29^ For example, ClassyFire^30^ allows the classification of known molecular structures based on a consistent ontology of chemical descriptors.

**Fig. 1:**
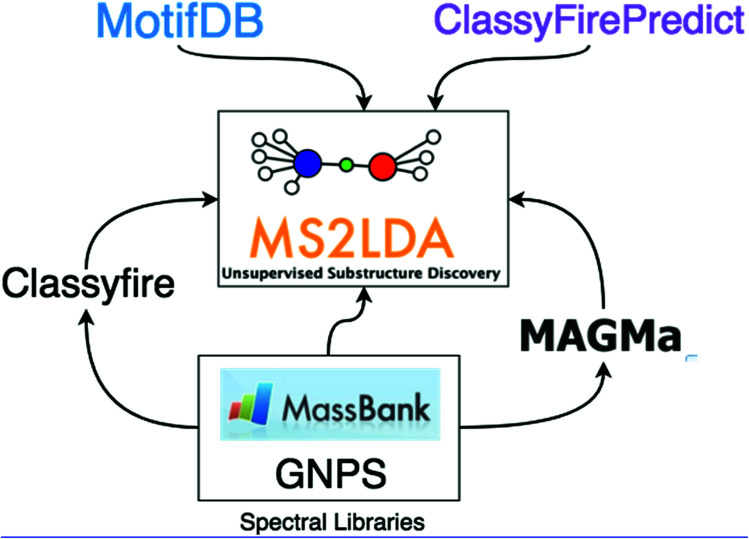
The extensions to the original MS2LDA model described in this paper. MotifDB provides a platform for storing and re-using annotated Mass2Motifs. MAGMa and ClassyFire are both used with standard datasets to predict substructures corresponding to Mass2Motif features, identify terms enriched within Mass2Motifs and provide insight into the structural makeup of the MassMotifs derived from them. ClassyFirePredict extends this idea to non-standard data by predicting ClassyFire terms directly from the mass spectra.

In this work, we demonstrate how the integration of both MAGMa and ClassyFire terms within the ms2lda.org application facilitates the structural characterisation of a larger number of discovered Mass2Motifs. The extensions to the original ms2lda.org platform presented here are shown schematically in Fig. 1. MAGMa is used for the automated annotation of mass and neutral loss features within Mass2Motifs discovered from reference spectra, using the known chemical structures as candidates. These Mass2Motifs can then be compared with Mass2Motifs discovered in other experiments, increasing annotation coverage.

ClassyFire terms are used in two ways. Firstly, Mass2Motifs derived from reference spectra are mined for terms enriched in the molecules in which the Mass2Motifs are present. This provides rich structural information about the Mass2Motifs, against which newly discovered Mass2Motifs can be queried. Secondly, using the terms from known reference spectra, we present a machine learning approach (ClassyFirePredict) that predicts terms for spectra from experimental data. Mass2Motifs derived from these experimental data can then be mined for enriched terms based upon the predictions. Using a publicly available annotated MS2LDA experiment, we show how this can guide the user for annotation of fragment-based Mass2Motifs such as flavonoid and saccharide related motifs. Both ClassyFire systems are available at ms2lda.org.

Finally, to effectively reuse previously annotated motifs, we introduce MotifDB (available from ms2lda.org).^31^ MotifDB stores annotated Mass2Motifs with their MS/MS features. A number of annotated Mass2Motif sets from various sources including plant extracts, urine, and standards, are already available for matching against Mass2Motifs discovered in new experiments.

We expect that the augmentations to the ms2lda.org web app will allow researchers to more rapidly decipher complex mixtures and create annotated and curated sets of Mass2Motifs. Those in turn will be effective in future experiments to more quickly assess the presence of specific molecular types in complex mixtures and assess the chemical diversity of those mixtures based on substructure recognition. We expect these substructure-based annotation strategies to become essential for deciphering complex mixtures and enabling meaningful biochemical interpretation.

## Methods

### Integrating ClassyFire substituent terms

ClassyFire terms were derived through the ClassyFire API for two of the public standard datasets (massbank_binned_005 and gnps_binned_005 – see Data availability section) stored within ms2lda.org using the ClassyFire web API^30^ based on the molecules’ InChIKeys. The substituent terms were stored in the database and linked to the relevant molecules such that they are visible when the molecule is explored. Additional functionality was added to ms2lda.org to summarize the terms within a particular Mass2Motif. In particular, based on actual values of the fragment spectra to Mass2Motif probability and overlap score thresholds outputted by MS2LDA,^32^ the molecules associated with each Mass2Motif are extracted, along with their ClassyFire substituent terms. For each term, the proportion of molecules associated with the Mass2Motif that include the term is computed, along with the proportion of molecules in the experiment. Comparing these terms provides evidence as to how unique and concentrated that term is in the Mass2Motif.

When working with new experimental data, exploring ClassyFire terms from standard molecules is useful if a discovered motif closely matches one of those in the standards experiments. To further extend this functionality, we have developed a machine learning approach that can predict putative ClassyFire terms from any mass spectrum. A multilayer neural network was produced that, for a binned mass spectrum, predicts the probability of the presence/absence for each ClassyFire term. The network was built in Python using Keras.^33^ Spectral data are currently binned into bins of width 1 Da, with *m*/*z* values over 1000 discarded. After normalizing so that the base bin (*i.e.* the most intense bin in a particular spectrum) had intensity of 1000.0, the data were log transformed (after adding 1.0 to avoid problems associated with taking the log of zero). The network consists of a 1000-dimensional densely-connected input layer, followed by two hidden dense layers (of dimension 500 and 200) and then an output layer with dimension equal to the number of ClassyFire substituent terms. Non-linear ReLU (rectified linear unit) activation functions were used for the hidden layers, and a sigmoid function was used for the output layer. The model was optimized using the binary cross entropy loss function. This model represents our initial network design and it is likely that it could be optimized further.

An initial training and validation phase was undertaken using a filtered dataset of 10 038 unique tandem mass spectra with associated chemical structures retrieved from Global Natural Products Social Molecular Networking (GNPS). This dataset was created as follows. First, all public libraries from GNPS were assembled. Subsequently, we used a script in Python (see Code availability section) to sub-select only tandem mass spectra with full chemical structural information in computer readable format (at least SMILES available) to create a dataset in the .MGF data format followed by the selection of 10 105 unique molecules based on the first 14 digits of the InChIKeys with precursor *m*/*z* < 1000. The ClassyFire API generated classifications for 10 038 of these molecules, resulting in the final dataset.

Ten random splits into training (90%) and validation (10%) were used to assess the performance with respect to each term. Within each split, the area under the receiver operating characteristic curve (AUC) was computed, and these were averaged across the ten splits. Based on this analysis, we selected 444 terms that could be reliably predicted for the final classifier. These 444 terms were chosen *via* two conditions: firstly, all terms with an average AUC across the ten splits of greater than 0.7, and also, terms with an AUC of between 0.6 and 0.7 that appeared in at least 0.5% of the molecules in the dataset. These additional terms were included to increase coverage under the assumption that some false positives can be tolerated for individual molecules, as they are likely to be filtered out when we explore terms at the Mass2Motif level. Finally, the model was re-trained using these 444 terms and all of the available training data.

The predictive model was incorporated into ms2lda.org, allowing users to assign putative ClassyFire terms to any molecules. These terms are then collated at the Mass2Motif level to aid in annotation in exactly the same manner as those linked to the reference molecules.

### MAGMa-MS2LDA integration

MAGMa was used to annotate Mass2Motif features as follows. All reference spectra for four data sets of known molecules that were subjected to MS2LDA (massbank_binned_005, gnps_binned_005, 2613 public spectra from various sources in positive ionization mode, and 551 public spectra in negative ionization mode from various sources – see Data availability section) were analyzed and annotated using MAGMa (see Code availability section). Each spectrum was annotated based upon its known structure resulting in the likely molecular substructures being assigned to individual peaks. Only the peaks used in the MS2LDA analysis were included in the MAGMa analysis, of which not all necessarily match with a simple substructure found within the reference molecule. Subsequently, the substructures were matched with the actual features used in the MS2LDA analysis (either fragments or losses within user-defined mass bins). For fragment features, the substructures assigned by MAGMa were stored both as a canonical SMILES, generated by the RDKit software library,^34^ and as a mapping (with atom indices) on the original molecule. A SMILES string was generated for the loss features by first removing the MAGMa substructure atoms from the complete molecule and generating a canonical SMILES from the remaining atoms. These SMILES may contain disconnected parts of the molecules (separated by a dot according the SMILES specifications). MAGMa substructure feature annotations were stored in MS2LDA and visualized in the web application with the ChemDoodle package.^35^

As a result, Mass2Motif pages in MS2LDA could now be augmented with the MAGMa substructure annotations as follows. For a given feature explained by a Mass2Motif, all substructures associated to the feature in the corresponding spectra are retrieved and grouped. It is possible that the same fragment or loss in two spectra could be assigned different molecular substructures by MAGMa, a consequence of different molecular structures having the same (or very similar) mass. For example, a methyl carboxylic acid or *O*-acetyl group could be assigned to a loss of 60.0225 depending upon the parent structure. For a particular Mass2Motif, all unique substructures are presented along with the number of times they occur in the corresponding spectra. Additionally, since the same binned fragment and neutral losses are used as global features across all experiments in MS2LDA.org, annotations for all (and new) features that have corresponding features in MAGMa-annotated experiments can be derived from the existing MAGMa annotations assigned to these shared global features. We show this new information in the Mass2Motif and Document pages of the ms2lda.org web app.

### MotifDB

Once Mass2Motifs have been annotated, it is useful to be able to search for them in future MS2LDA experiments. To this end, we have created a new application within MS2LDA.org called MotifDB: a database for annotated Mass2Motifs (http://ms2lda.org/motifdb). This database can be accessed *via* an API as well as being searchable against other experiments in the ms2lda.org web app. In particular, when an experiment has been run through MS2LDA.org, the user can start a motif matching procedure against Mass2Motifs stored in MotifDB. Where a Mass2Motif discovered in a new experiment exceeds a cosine similarity threshold with a Mass2Motif from MotifDB, the experimental motif can be linked to the MotifDB motif. The MotifDB annotation will now be highlighted in visualizations. It is important to realize that differential fragmentation mechanisms and different choices of collision energies between platforms can result in different fragmentation spectra.^36^ As a result, similar substructures discovered in data obtained from different mass spectrometry platforms (*i.e.*, quadrupole time-of-flight, orbitrap, and ion trap) could result in different Mass2Motifs that would still represent the same substructure information. However, as we^8^ and others^37^ have shown, there are many situations where substructures are represented by diagnostic mass features formed across different platforms or where molecules do have comparable fragmentation spectra. As MotifDB grows by community efforts, more and more Mass2Motifs learnt in experiments under different experimental conditions will be annotated and available to be matched against in the MotifDB database, allowing for more rapid characterization of diverse chemical mixtures.

## Code availability

The Python script to generate MAGMa annotations of standards datasets is provided on Github: https://github.com/iomega/motif_annotation.

The Python script to collect all GNPS library molecules including full metadata in .MGF format is provided on GitHub: https://github.com/madeleineernst/EditMGF/blob/master/CompileGNPSMGF_withInChIKey.py for which the following GNPS jobs are needed: https://gnps.ucsd.edu/ProteoSAFe/status.jsp?task=6e22f85aeb0744208e872d1640f508d9, https://gnps.ucsd.edu/ProteoSAFe/status.jsp?task=03fba62d93cb4cbfa3f72106d18f7d2c.

The scripts to prepare the GNPS library molecules for neural networking and perform the neural networking are provided on Github: https://github.com/sdrogers/nnpredict.

The code to perform MS2LDA is available at: https://github.com/sdrogers/lda.

The code for the ms2lda.org visualisation platform is available at: https://github.com/sdrogers/ms2ldaviz.

## Results

### MAGMa-based annotation of Mass2Motifs

#### MAGMa-MS2LDA annotations for previously analyzed Mass2Motifs

**Fig. 2:**
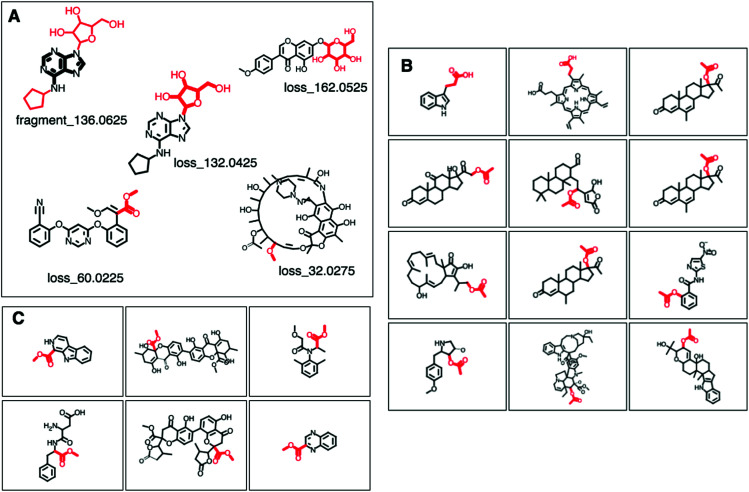
(A–C) Screenshots of the ms2lda.org web app with (A) MAGMa annotations of Mass2Motif features in 5 motifs discussed in the results section. Annotated fragments are highlighted in black and bold, whereas annotated losses are depicted in red and bold. (B) 12 examples of the 38 molecules for which the loss_60.0225 in GNPS Mass2Motif 49 was annotated with loss (CC(=O)O) in SMILES. (C) 6 examples of the 25 molecules for which the structurally related COC=O loss in SMILES was annotated for the same loss feature in GNPS Mass2Motif 49.

The integration of MAGMa with MS2LDA resulted in reference MS/MS MS2LDA experiments enriched with available MAGMa annotations for mass fragments and neutral losses for each fragmented molecule (Fig. 2A). MAGMa annotations were evaluated to identify how well they matched with previously (manually) annotated and validated motifs.^8^ For example, motif 59 in the GNPS reference set was manually annotated and validated to be related to the phenylalanine minus CHOOH fragment substructure (http://ms2lda.org/basicviz/view_parents/58316/). Indeed, for 79 out of 117 molecules exactly this substructure was annotated by MAGMa for mass fragment 120.0825, with confirmation for the related aromatic fragment 103.0525 for 29 out of 35 appearances. This indicates that indeed this motif is related to [phenylalanine minus CHOOH]; moreover, the MAGMa annotations also provide quick insight in structurally less related molecules in the motif that are included due to isomeric fragments giving rise to the same mass fragment. This highlights the need for manual validation of fragmentation patterns in molecules, which is now supported in the ms2lda.org web application.

Another example is the indole related GNPS motif 25 (http://ms2lda.org/basicviz/view_parents/58017/); here, for 47 out of 110 molecules, MAGMa annotated the 130.0675 mass fragment with a methylindole substructure, and for 11 out of 28 molecules, the 118.0675 mass fragment was annotated with the indole substructure. Interestingly, the MAGMa annotations facilitated insight in other isomeric substructures within this motif; for example, MAGMa annotated the 130.0675 fragment for 17 molecules with a 2-aminopropyl-phenyl substructure and for 6 molecules the related 2-aminoethyl-phenyl substructure, indicating that motif 25 is also associated to this aromatic substructure. Other annotations for the 130.0675 fragment included two isobaric substructures with a different elemental formula, the mass of which fell within the 0.005 Da mass bin.

**Fig. 3:**
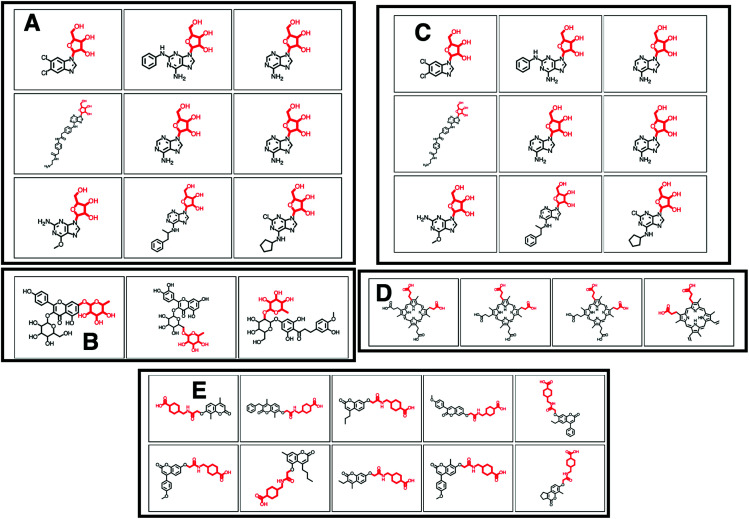
(A–E) Screenshots of the ms2lda.org web app with (A) 9 different molecules out of the 50 molecules that MAGMa annotated with a hexose moiety for the loss feature in MassBank Mass2Motif 41. (B) 3 examples of the 13 molecules where MAGMa annotated the loss feature in MassBank Mass2Motif 41 with a deoxyhexose moiety. (C) 9 out of the 27 molecules for which MAGMa annotated a pentose moiety for the loss feature in GNPS Mass2Motif 44. (D) Alternative loss annotation of the loss feature in GNPS Mass2Motif 44. (E) Oxyacetyl-amino-methyl-cyclohexane-1-carboxylic acid loss annotated in 10 molecules of GNPS Mass2Motif 439.

MAGMa also annotated neutral loss-based Mass2Motifs. For example, GNPS Mass2Motif 49 was previously annotated with “Loss possibly indicative of carboxylic acid group with 1-carbon attached” http://ms2lda.org/basicviz/view_parents/58174/. This annotation was confirmed by MAGMa with the loss being annotated as CC(=O)O (in SMILES) in 38 molecules out of 132 (12 of which can be seen in Fig. 2B). 25 of the remaining molecules were annotated with the structurally related COC=O loss (Fig. 2C) and the remainder of the molecules with other isomeric losses. A similar example can be found in the MAGMa annotations for GNPS motif 18 http://ms2lda.org/basicviz/view_parents/58383/ annotated as acetyl loss, as can be seen here: http://ms2lda.org/basicviz/show_doc/273058/. Furthermore, for Massbank Mass2Motif 41, “Loss indicative of [hexose minus H20]” the majority of the MAGMa-annotated losses (50 out of 64) were glucose related http://ms2lda.org/basicviz/view_parents/57676/ (Fig. 3A) with 13 being deoxyhexose moieties (Fig. 3B) that – unusually – included the connecting oxygen atom upon fragmentation of the main scaffold, which normally remains connected to the main scaffold. In the case of GNPS Mass2Motif 44, “[Pentose (C5-sugar)-H_2_O] related loss – indicative for conjugated pentose sugar”, MAGMa confirmed the pentose loss for 27 out of 56 molecules (Fig. 3C) http://ms2lda.org/basicviz/view_parents/58179/. For this motif, alternative loss annotations were also annotated by MAGMa, as shown in Fig. 3D.

Finally, GNPS motif 54 was annotated as ferulic acid related http://ms2lda.org/basicviz/view_parents/58325/. The MAGMa annotations show how important it is for this motif that the four mass fragments are all present, since 73 molecules contained mass fragment 177.0525. whereas for mass fragment 117.0325. 14 out of 19 molecules contained ferulic acid related substructures. Thus, whereas all GNPS Mass2Motif 54 related fragments have isomeric substructures unrelated to ferulic acid, their combined presence is highly indicative of the presence of ferulic acid.

#### MAGMa-MS2LDA integration for annotation of yet unexplored Mass2Motifs

In addition to previously annotated motifs, MAGMa annotations of not yet explored Mass2Motifs were analyzed. Fig. 2A shows MAGMa annotations for Mass2Motif fragment and loss features for five of the here described motifs in one of their related molecules. For example, GNPS Mass2Motif 152 could now be easily annotated as methanol loss resulting from the presence of a methoxy group http://ms2lda.org/basicviz/view_parents/58033/. The methoxy related loss could be annotated in 51 out of 58 molecules by MAGMa. Another methoxy group related GNPS Mass2Motif (374) was uncovered, where the loss of 16.0325 was assigned to CH_4_ in 33 out the 38 molecules in the motif. In addition, GNPS Mass2Motif 188 could be annotated as related to a 2-dimethylamine-ethanol loss (*m*/*z* 89.0825), which was present in 9 out of the 14 molecules http://ms2lda.org/basicviz/view_parents/58098/. Other examples where MAGMa facilitated motif annotations include MassBank Mass2Motif 315 (benzyl and phenoxy group containing molecules), where for 77 out of the 84 associated molecules, the benzyl moiety was annotated by MAGMa. Moreover, in 20 molecules the phenoxy group was annotated for the motif fragment *m*/*z* 95.0475; however, interestingly, in 34 cases this fragment was present in the MS/MS spectrum, while there was no phenoxy group present in the corresponding reference molecule, nor was there any other substructure that could be assigned to this fragment. A possible explanation is that rearrangements are taking place in the mass spectrometer during the fragmentation process leading to the formation of phenoxy fragments as all these molecules do contain benzyl moieties. Here, the MAGMa-MS2LDA integration provides quick insight in assessing the consistency of structural annotations based on the presence/absence of mass fragments. Furthermore, MassBank Mass2Motif 443 could be annotated as “aniline related” due to the fact that 30 of the 32 associated molecules contained an aniline or substituted aniline substructure annotated by MAGMa http://ms2lda.org/basicviz/view_parents/57561/. Finally, GNPS Mass2Motif 439 (http://ms2lda.org/basicviz/view_parents/57921/) was shown by the MAGMa annotation to originate from a specific series of oxyacetyl-amino-methyl-cyclohexane-1-carboxylic acids with a characteristic series of losses (Fig. 3E). Based on the above examples, we show how MAGMa annotations are very helpful during the Mass2Motif annotation process. Our manual analysis of neutral losses is hampered by our inability to detect these generally smaller losses rather than larger scaffolds, which are easier to recognize – and MAGMa annotations are particular helpful here.

#### Chemical classification-based annotation of Mass2Motifs from standards

With increasing numbers of library MS/MS spectra available, the number of Mass2Motifs that can be extracted from those spectra will steadily increase. An alternative to the MAGMa substructure annotations for annotating this growing number of Mass2Motifs is the use of chemical classification. ClassyFire substituent terms for all of the molecules in the reference MS/MS data set were collected.^30^ These substituent terms are based upon more than 5000 SMARTS patterns and are typically used by ClassyFire to organise molecules into a hierarchical chemical ontology. Here, we combined the substituent terms associated with molecules to look for terms that are enriched within Mass2Motifs with respect to their presence across the entire data set. For example, GNPS Mass2Motif 43 was previously annotated as being related to the adenine core structure http://ms2lda.org/basicviz/view_parents/58177/. The enriched substituent terms clearly correlate with this previous annotation: terms like *aminopyrimidine* and *6-aminopurine* are enriched (present in 64.3% and 52.4% of the molecules associated with this Mass2Motif, respectively) as compared to their percentage of occurrence in the entire GNPS data set (2.3% and 0.6%, respectively) (ESI Table S1†). In addition, GNPS Mass2Motif 72 was enriched with *amine* and *tertiary amine* terms (58.3% and 45.2% within the motif, 25% and 14.6% across the experiment), which is consistent with its annotation as diethylamino or dimethylaminoethyl substructure (ESI Table S2†). GNPS Mass2Motif 1 was enriched with oxosteroid related substituent terms *oxosteroid* and *3-oxosteroid* (present at 45.6% and 44.4% within the motif, and 3.9% and 3.3% across the experiment) matching its previous annotation as “sterone related” http://ms2lda.org/basicviz/view_parents/58328/.

**Fig. 4:**
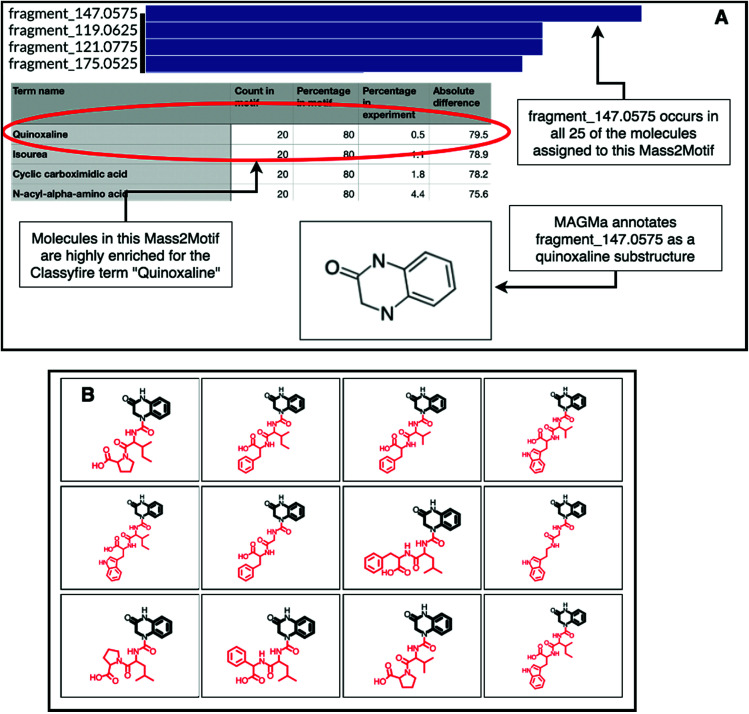
(A) Top: feature frequency plot for GNPS Mass2Motif 60; middle: most enriched ClassyFire substituent terms in the same motif; bottom: MAGMa assigned the quinazolinol substructure in 22 of the 25 molecules associated to this motif. (B) Screenshot of the ms2lda.org web app with the MAGMa annotated quinazolinol substructure highlighted in 12 of the 22 molecules.

The natural product substructure of quinazolinol (4-quinazolinone) was previously assigned to GNPS Mass2Motif 60 http://ms2lda.org/basicviz/view_parents/57956/. Demonstrating the power of the combination of MAGMa and ClassyFire, MAGMa annotated the quinoxaline substructure in 22 out of the 25 molecules (Fig. 4) and the enriched ClassyFire terms confirm this annotation (the *quinoxaline* term is present in 39.2% of molecules within the motif *versus* 0.5% of molecules within the experiment). This example shows that collected substituent terms can be used as guidance for Mass2Motif annotations in reference MS/MS data sets thereby providing consistent and widely-used chemical ontology terms.

With help of MAGMa and ClassyFire a number of novel annotations were made. For example, GNPS Mass2Motif 6 was annotated with the diphenyl-containing substructure following MAGMa annotations for its mass features and its enriched ClassyFire terms http://ms2lda.org/basicviz/view_parents/58331/ (Table 1). The MAGMa annotations of a methoxy group in GNPS Mass2Motif 152 matched with corresponding ClassyFire terms being enriched in this motif, such as *methyl ester* and *carboxylic acid ester* (Table 2). This is remarkable for such a small substructure. Interestingly, for GNPS Mass2Motif 439 (Fig. 3E), amongst the substituent terms ClassyFire did return, there were no helpful terms for Mass2Motif annotation, whilst MAGMa could annotate relevant substructures to guide Mass2Motif annotation, indicating the complementarity of these approaches. Overall, the enriched chemical classification terms confirmed and strengthened the manual and MAGMa annotations, and as such they may support and promote the use of consistent chemical terminology during the annotation process.

**Table 1:** Top 10 most enriched ClassyFire substituent terms for GNPS Mass2Motif 6, which could in this study be annotated as diphenyl substructure related. The term name represents the ClassyFire substituent term, the count in motif is the number of times the term appeared in a molecule associated to the Mass2Motif, the percentage in motif is the percentage of the count in motif over the total number of molecules in the motif, the percentage in experiment is the percentage of the number of term occurrences in molecules within the entire experiment over the total number of molecules, and the absolute difference is the absolute difference between the two percentages

Term name	Count in motif	Percentage in motif	Percentage in experiment	Absolute difference
Diphenylmethane	23	52.3	2.1	50.2
Tertiary aliphatic amine	21	47.7	13.7	34
Tertiary amine	21	47.7	14.6	33.2
Amine	24	54.5	25	29.5
Heteroaromatic compound	5	11.4	36.8	25.4
Aromatic heteropolycyclic compound	7	15.9	40.3	24.4
Benzenoid	10	22.7	45	22.3
Aromatic homomonocyclic compound	14	31.8	9.6	22.2
Benzylether	8	18.2	0.6	17.5
Dialkyl ether	11	25	7.7	17.3

**Table 2:** Top 10 most enriched ClassyFire substituent terms for GNPS Mass2Motif 152 that was annotated with the help of MAGMa as methoxy group related. The term name represents the ClassyFire substituent term, the count in motif is the number of times the term appeared in a molecule associated to the Mass2Motif, the percentage in motif is the percentage of the count in motif over the total number of molecules in the motif, the percentage in experiment is the percentage of the number of term occurrences in molecules within the entire experiment over the total number of molecules, and the absolute difference is the absolute difference between the two percentages

Term name	Count in motif	Percentage in motif	Percentage in experiment	Absolute difference
Methyl ester	14	24.1	2.3	21.8
Carboxylic acid ester	20	34.5	13.9	20.6
Dialkyl ether	15	25.9	7.7	18.2
Enoate ester	11	19	2.9	16.1
Alpha,beta-unsaturated carboxylic ester	11	19	2.9	16.1
Ether	26	44.8	30.9	13.9
Dihydropyridinecarboxylic acid derivative	6	10.3	0.6	9.8
Carboxylic acid	2	3.4	13.3	9.8
Enamine	5	8.6	0.6	8.1
Monocarboxylic acid or derivatives	16	27.6	19.7	7.9

#### Chemical classification-based annotations of Mass2Motifs from non-standards

Using more than 10 000 unique GNPS Library reference MS/MS spectra, a neural network was trained to infer 444 ClassyFire substituent terms from fragmentation data (ClassyFirePredict). To evaluate the predictive model, it was applied to a public MS2LDA experiment of 71 Rhamnaceae plant extracts (see Data availability section) in which more than 20 motifs had previously been manually annotated.^14^ Terms predicted for each spectrum were collected at the Mass2Motif level and compared with the manual annotations. Rhamnaceae Mass2Motif 33 had been manually annotated with a xylose or arabinose saccharide moiety. The ClassyFire predictions indicated enrichment of *alcohol* and *secondary alcohol* terms as well as *glycosyl* and *O-glycosyl compounds* which are all saccharide related terms http://ms2lda.org/basicviz/view_parents/109416/. Thus, the ClassyFirePredict and manual annotations correspond well for this Mass2Motif, indicating that ClassyFirePredict can assist in Mass2Motif annotations. The unannotated Rhamnaceae Mass2Motif 196 was enriched with overlapping saccharide-related terms, which suggests that this is also a saccharide related motif http://ms2lda.org/basicviz/view_parents/109504/. Rhamnaceae Mass2Motifs 3 and 86 were annotated with the 3-hydroxyflavanoid cores myricetin and quercetin, respectively http://ms2lda.org/basicviz/view_parents/109575/ and http://ms2lda.org/basicviz/view_parents/109460/. Indeed, the predicted enriched ClassyFire terms clearly point to flavonoid related terms like *chromone* and *phenol*, which is also reflective of their presence in the training data. Finally, Rhamnaceae Mass2Motif 148 was annotated as a cyclopeptide alkaloid related motif http://ms2lda.org/basicviz/view_parents/109419/. Motif members were previously structurally annotated and found to be cyclic peptides sharing a benzenoid moiety (https://gnps.ucsd.edu/ProteoSAFe/gnpslibraryspectrum.jsp?SpectrumID=CCMSLIB00004679280#%7B%7D).^14^ The predicted enriched ClassyFire terms reflect these cyclopeptidic structures well. In particular, *benzenoid* is highly enriched (85.7% present in the motif *versus* 18.5% in the experiment), as is *organonitrogen compound* (60.7% in motif *versus* 29.8% in experiment). Thus, we conclude that ClassyFirePredict can provide annotations that are useful annotations in guiding the analysis of Mass2Motifs from experimental data.

#### MotifDB

**Fig. 5:**
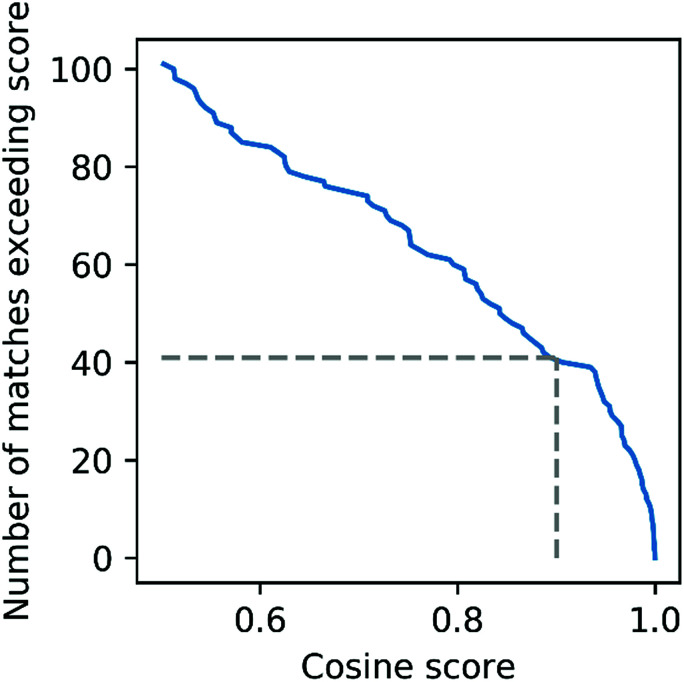
Distribution of Mass2Motif matching scores for a urine dataset matched against the urine MotifSet in MotifDB. The dashed line shows the number of Mass2Motifs (41) that could be matched against the MotifSet with a cosine score of 0.9 or more.

The new motif matching pipeline was used to match newly discovered Mass2Motifs in 5021 mass spectra from a publicly available human urine sample with a set of Mass2Motifs previously manually annotated from urine samples of the same cohort run under the same experimental conditions (http://ms2lda.org/basicviz/manage_motif_matches/709/).^32^ Of the 300 Mass2Motifs discovered, 102 could be matched against 82 unique Mass2Motifs from MotifDB with cosine scores of 0.5 or greater, of which 41 had cosine scores greater than 0.9. The distribution of scores is shown in Fig. 5. The ten highest scoring matches are shown in ESI Table S3† along with the annotation and the number of molecules that are assigned to the discovered motif (at a probability threshold of 0.1 and an overlap threshold of 0.3). These matches include Mass2Motifs related to urine-related substructures such as creatine and carnitine that have large degrees in the 5021 mass spectra, indicating that these substructures are abundant in urine as they are present in many fragmented molecules. In total, across the 102 matched motifs, 3715 unique molecules include at least one of the 102 matched Mass2Motifs (out of a total of 5021 in the experiment; 74%) and 2879 (57%) unique molecules include at least one Mass2Motif matched with a score of >0.9. These percentages indicate the potential of annotating complex mixtures through substructure assignments.

To further evaluate the power of motif matching against MotifDB we compared the urine motif set from MotifDB with Mass2Motifs discovered in fragmentation spectra of 6 urine samples from a different cohort analysed under the same experimental conditions (http://ms2lda.org/basicviz/manage_motif_matches/601/).^22^ In this case, of the 200 Mass2Motifs, 55 could be matched at a threshold of at least 0.5 (covering 573 of the 1163 molecules; 49%) and 20 at a threshold of 0.9 (404 molecules; 35%). Although, as expected, the number of matches is lower than in the first example, the ability to immediately match approximately a quarter of the discovered motifs (allowing some level of annotation for half of the molecules) highlights the generalizability of Mass2Motifs across sample sets. This approach aids the discovery and prioritization of novel Mass2Motifs that may well represent xenobiotic-related chemistry (*i.e.*, drugs, food, *etc.*) not previously encountered.

## Conclusions and future outlook

In this paper, we have described multiple extensions to the MS2LDA platform (all implemented on the ms2lda.org web app) that enhance the ability of analysts to characterize the makeup of complex mixtures of metabolites. The extensions all make it easier to characterize the Mass2Motifs onto which MS2LDA allows experimental data to be decomposed. These Mass2Motifs often represent chemical substructures and annotating them allows some degree of annotation to all MS2 spectra that include them, as often a relatively small number of annotated Mass2Motifs provide information about a significant proportion of the molecules in an experiment.^8^

The extensions move the platform forward in two general directions. The first, MotifDB, provides a platform that allows for the storage of annotated Mass2Motifs that can then be accessed *via* an API (details at http://ms2lda.org/motifdb) or used within ms2lda.org by allowing users to match Mass2Motifs discovered within their experiments to those stored in MotifDB. In our experiments with human urine data, we found that roughly 25% of the Mass2Motifs in a urine dataset from a different cohort than the dataset from which the annotated motifs were generated could be matched against Mass2Motifs from MotifDB. These 25% of Mass2Motifs were associated to about 50% of the molecules.

The second direction is the collation of known and predicted molecular properties for individual molecules across Mass2Motifs. Here, we have presented three advances. Firstly, the use of MAGMa on databases of standards that had been analysed with MS2LDA to annotate their fragment spectra with substructures. We show how MAGMa-Mass2Motif annotations provide quick insight in ambiguity of annotations in case of isomeric substructures. These substructure annotations can then be propagated to the features in the Mass2Motifs, providing relevant insight into the substructures they could represent.

The second advance propagates the ClassyFire substituent terms for the same datasets of chemical standards to the Mass2Motif level. Finally, for “unknown” molecules measured in experimental data, we have introduced a machine learning approach based on a neural network that can predict a subset of ClassyFire substituent terms from the spectral data. This model has some limitations: (i) the predictive power is dependent on the chemical diversity present in available training spectra, (ii) the current training set consists of series of structurally correlated molecules, and (iii) very small substructures will be difficult to predict due to their usually widespread presence in molecules with structurally diverse larger scaffolds, making it harder to recognize the specific chemical terms connected to these smaller substructures. Nevertheless, we show that for fragment-based Mass2Motifs from complex mixtures, the predicted terms can guide Mass2Motif annotations. Again, these can be propagated to the Mass2Motif level, providing insight into their structural makeup. We foresee that by annotating more and more Mass2Motifs, the metabolite annotation of yet unknown molecules in complex mixtures – the main bottleneck in untargeted metabolomics data analysis – will become easier. The proposed machine learning approach has the potential for further exploration and optimization. The model can be further augmented by inclusion of neutral loss features as well as mass shifts, which are expected to improve chemical predictions for loss-based motifs such as loss of hexose or deoxyhexose and amino acid related motifs, respectively.

As more Mass2Motifs are extracted and annotated from the growing datasets of standards, MotifDB will grow and the coverage across experiments will increase. We also foresee users including annotated motif sets within their LDA experiment, thereby simultaneously finding known substructure patterns and discovering new ones with the benefit of combining supervised and unsupervised motif discovery in one analysis. Furthermore, users would then also be able to decompose single spectra over these motif sets through an API.

The MAGMa and ClassyFire based annotations can significantly enhance the process of annotation of the rapidly growing (number of) datasets and Mass2Motifs. The expected growth in available fully annotated reference spectra will also increase the training sets available for our ClassyFire predictor, increasing performance and increasing the set of terms that we can confidently predict. Furthermore, the implementation of chemical ontology from ClassyFire assists in more consistent annotations of motifs by using chemical terminology from an ontology.

We expect that substructure-based annotation strategies will prove to be essential to decipher complex mixtures and enable meaningful biochemical interpretation. Our work represents key steps of this workflow by recognizing mass spectral patterns, semi-automated structural annotation and storage of them. An increasing amount of structurally annotated Mass2Motifs will allow metabolomics researchers to gain some structural information on the majority of fragmented molecules. The further closing of the structural annotation gap in metabolomics will make untargeted metabolomics a very powerful tool for studying complex mixtures.

## Author contributions

SR, LR, and JJJvdH conceptualized the study. LR and JW designed and implemented MAGMa-MS2LDA integration. ME extracted well-annotated publicly available spectral data from GNPS. SR and JJJvdH designed ClassyFire predictions. CWO and SR built the neural network model for ClassyFire predictions and SR integrated it within MS2LDA. CWO, LR, SR, and JJJvdH analyzed data. All authors contributed to the writing of the manuscript and agreed on the content.

## Funding

JJJvdH is supported by an ASDI eScience grant (ASDI.2017.030) from the Netherlands eScience Center (NLeSC). SR is supported by an BBSRC grant BB/R022054/1 and a Carnegie Trust for Scotland grant.

## Conflicts of interest

The authors declare there are no conflicts of interest.

## Supplementary Material

FD-218-C8FD00235E-s001

## Data Availability

The following public MS2LDA experiments were used in this manuscript. Reference molecule data sets: massbank_binned_005 – http://ms2lda.org/basicviz/show_docs/190/. Gnps_binned_005 – http://ms2lda.org/basicviz/show_docs/191/. 2613 public spectra from various sources in positive ionization mode – http://ms2lda.org/basicviz/summary/304/. 551 public spectra in negative ionization mode from various sources – http://ms2lda.org/basicviz/summary/305/. Complex mixtures: Urine38_POS_mzML_standardLDA_005binned – http://ms2lda.org/basicviz/summary/709. UrineDrugs_MolNetw_WorkshopSeattle2018 – http://ms2lda.org/basicviz/summary/601/. Rhamnaceae_plant_extracts_KyoBin_200Motifs_MS1_peaktable – http://ms2lda.org/basicviz/summary/566/.
