# Supplementary material for: Deciphering complex metabolite mixtures by unsupervised and supervised substructure discovery and semi-automated annotation from MS/MS spectra
Source: Faraday Discuss. 2019 Jan 29;218:284–302. doi: 10.1039/c8fd00235e (PMC13250438; doi:10.1039/c8fd00235e)
Supplement: FD-218-C8FD00235E-s001 [file FD-218-C8FD00235E-s001.pdf]

Electronic Supplementary Material (ESI) for Faraday Discussions.
This journal is © The Royal Society of Chemistry 2019

Supplementary information to:

Deciphering complex metabolite mixtures by unsupervised and supervised substructure discovery and semi-automated annotation from MS/MS spectra

Simon Rogers[1], Cher Wei Ong[1], Joe Wandy[2], Madeleine Ernst[3,4], Lars Ridder[5], Justin J.J. van der Hooft[6*]

1) School of Computing Science, University of Glasgow, Glasgow, UK
2) Glasgow Polyomics, University of Glasgow, Glasgow, UK
3) Collaborative Mass Spectrometry Innovation Center, Skaggs School of Pharmacy and Pharmaceutical Sciences, University of California, San Diego, La Jolla, CA, USA.
4) Skaggs School of Pharmacy and Pharmaceutical Sciences, University of California, San Diego, San Diego, California, USA
5) Netherlands eScience Center, Amsterdam, The Netherlands
6) Bioinformatics Group, Wageningen University, Wageningen, The Netherlands
*corresponding author: justin.vanderhooft@wur.nl

Contents:

Supplementary Table S1

Supplementary Table S2

Supplementary Table S3

Table S1: Top 10 most enriched ClassyFire substituent terms for GNPS Mass2Motif 43 previously annotated as adenine related. Term name represents the ClassyFire substituent term, Count in motif is the number of times the term appeared in a molecule associated to the Mass2Motif, Percentage in motif is the percentage of the count in motif over the total number of molecules in the motif, Percentage in experiment is the percentage of the number of term occurrences in molecules within the entire experiment over the total number of molecules, and Absolute difference is the absolute difference between the two percentages.

Term name	Count in motif	Percentage in motif	Percentage in experiment	Absolute difference
**Aminopyrimidine**	27	64.3	2.3	62
**Imidazole**	27	64.3	4	60.2
**Pyrimidine**	27	64.3	4.5	59.8
**Azole**	27	64.3	8.1	56.2
**Imidolactam**	25	59.5	3.8	55.7
**N-substituted imidazole**	24	57.1	3.2	53.9
**6-aminopurine**	22	52.4	0.6	51.8
**Purine**	21	50	0.7	49.3
**Imidazopyrimidine**	19	45.2	0.7	44.5
**N-glycosyl compound**	19	45.2	0.8	44.4

Table S2: ClassyFire substituent terms for GNPS Mass2Motif 72 annotated as diethylamino or dimethylaminoethyl substructure related. Term name represents the ClassyFire substituent term, Count in motif is the number of times the term appeared in a molecule associated to the Mass2Motif, Percentage in motif is the percentage of the count in motif over the total number of molecules in the motif, Percentage in experiment is the percentage of the number of term occurrences in molecules within the entire experiment over the total number of molecules, and Absolute difference is the absolute difference between the two percentages.

Term name	Count in motif	Percentage in motif	Percentage in experiment	Absolute difference
**Amine**	49	58.3	25	33.3
**Organoheterocyclic compound**	6	7.1	38	30.9
**Tertiary amine**	38	45.2	14.6	30.7
**Tertiary aliphatic amine**	37	44	13.7	30.3

Table S3: Then highest scoring matches of Mass2Motifs discovered from urine matched against a set of urine-derived Mass2Motifs in MotifDB. Motif is the experimental Mass2Motif, MotifDB Motif is the matched motif from MotifDB, Score is the cosine similarity score between the two Mass2Motifs, MotifDB Annotation the structural annotation from MotifDB, and the Number of molecules are the number of molecules associated with the experimental Mass2Motif (out of 5021 in total).

Motif	MotifDB Motif	Score	MotifDB Annotation	Number of molecules
motif_25	urine_mass2motif_286.m2m	1.000	C4H8N based Mass2Motif - indicative for proline arginine ornitine citrulline and N-containing ring structures	217
motif_181	urine_mass2motif_233.m2m	1.000	Loss of 60.0248 - unclear what it points to	86
motif_49	urine_mass2motif_194.m2m	0.999	Creatinine related Mass2Motif	154
motif_114	urine_mass2motif_126.m2m	0.999	C7H7 and C5H5 fragments - indicative of methylbenzene substructure (aromatic)	75
motif_193	urine_mass2motif_273.m2m	0.999	Fragments (C4H8NO ring fragment - and C4H7O2 and C4H5O fragments) indicative for C4H10NO2 amino acid substructure	40
motif_206	urine_mass2motif_230.m2m	0.999	Water loss - indicative of a free hydroxyl group	147
motif_238	urine_mass2motif_18.m2m	0.999	C2H3N loss - could be specific for a type of ring?	130
motif_151	urine_mass2motif_293.m2m	0.998	Carnitine related Mass2Motif ,acylcarnitines are prevalent	419
motif_68	urine_mass2motif_228.m2m	0.997	Lysine related Mass2Motif	191
motif_72	urine_mass2motif_27.m2m	0.996	C5H10NO and C3H6N fragments - most likely N-methyl-morpholine substructure	138
